# Immune Response-Dependent Assembly of IMP Dehydrogenase Filaments

**DOI:** 10.3389/fimmu.2018.02789

**Published:** 2018-11-29

**Authors:** S. John Calise, Georges Abboud, Hideko Kasahara, Laurence Morel, Edward K. L. Chan

**Affiliations:** ^1^Department of Oral Biology, University of Florida, Gainesville, FL, United States; ^2^Department of Pathology, Immunology, and Laboratory Medicine, University of Florida, Gainesville, FL, United States; ^3^Department of Physiology and Functional Genomics, University of Florida, Gainesville, FL, United States

**Keywords:** cell proliferation, cytoophidia, enzyme polymerization, IMPDH, lymphocytes, purine synthesis, rods and rings, T cell activation

## Abstract

Inosine monophosphate dehydrogenase (IMPDH) catalyzes the conversion of IMP to xanthosine monophosphate, the rate-limiting step in *de novo* guanosine monophosphate (GMP) synthesis. In cultured cells, IMPDH polymerizes into micron-scale filamentous structures when GMP synthesis is inhibited by depletion of purine precursors or by various drugs, including mycophenolic acid, ribavirin, and methotrexate. IMPDH filaments also spontaneously form in undifferentiated mouse embryonic stem cells and induced pluripotent stem cells, hinting they might function in various highly proliferative cell types. Therefore, we investigated IMPDH filament formation in human and murine T cells, which rely heavily on *de novo* guanine nucleotide synthesis to rapidly proliferate in response to antigenic challenge. We discovered extensive *in vivo* IMPDH filament formation in mature T cells, B cells, and other proliferating splenocytes of normal, adult B6 mice. Both cortical and medullary thymocytes in young and old mice also showed considerable assembly of IMPDH filaments. We then stimulated primary human peripheral blood mononuclear cells *ex vivo* with T cell mitogens phytohemagglutinin (PHA), concanavalin A (ConA), or antibodies to CD3 and CD28 for 72 h. We detected IMPDH filaments in 40–60% of T cells after activation compared to 0–10% of unstimulated T cells. Staining of activated T cells for the proliferation marker Ki-67 also showed an association between IMPDH filament formation and proliferation. Additionally, we transferred ovalbumin-specific CD4^+^ T cells from B6.OT-II mice into B6.Ly5a recipient mice, challenged these mice with ovalbumin, and harvested spleens 6 days later. In these spleens, we identified abundant IMPDH filaments in transferred T cells by immunofluorescence, indicating that IMPDH also polymerizes during *in vivo* antigen-specific T cell activation. Overall, our data indicate that IMPDH filament formation is a novel aspect of T cell activation and proliferation, and that filaments might be useful morphological markers for T cell activation. The data also suggest that *in vivo* IMPDH filament formation could be occurring in a variety of proliferating cell types throughout the body. We propose that T cell activation will be a valuable model for future experiments probing the molecular mechanisms that drive IMPDH polymerization, as well as how IMPDH filament formation affects cell function.

## Introduction

The ability of various enzymes to polymerize into higher-order structures has been demonstrated over the last few decades (1). Classic examples include acetyl-CoA carboxylase (2), glutamate dehydrogenase (3), and glutamine synthetase (4). More recently, unbiased screens have established that formation of reversible polymers or other assemblies by metabolic enzymes is more common than previously thought (5–7). The polymerization of two enzymes in *de novo* nucleotide biosynthesis, cytidine triphosphate synthase (CTPS) and inosine monophosphate dehydrogenase (IMPDH), has been of increasing interest, in particular. CTPS catalyzes the rate-limiting step in *de novo* CTP biosynthesis and polymerizes into micron-scale filaments in species of bacteria, budding yeast, fruit flies, and mammalian cells (5, 8, 9). Polymerization regulates the catalytic activity of CTPS (10–12), acetyl-CoA carboxylase (13), and glutamine synthetase (14), but its function is less clear for many enzymes, including IMPDH.

IMPDH catalyzes the rate-limiting step in *de novo* guanosine monophosphate (GMP) synthesis, the NAD^+^-dependent oxidation of IMP into xanthosine monophosphate, which is then converted into GMP by GMP synthase. In humans, two genes encode IMPDH1 and IMPDH2, which have similar catalytic activity and share 84% amino acid sequence identity (15, 16). In general, IMPDH1 is constitutively expressed at low levels in most tissues, but is high in retina, spleen, and resting peripheral blood mononuclear cells (PBMCs), while IMPDH2 is upregulated during proliferation and transformation (17–19). Like the two CTPS isoforms, both IMPDH isoforms can assemble into micron-scale filaments, also referred to as “rods and rings” structures, in mammalian cells (20–22). These filaments appear to be bundles of interacting apolar, helical polymers composed of stacked IMPDH octamers (23–25). Allosteric binding of adenine and guanine nucleotides at the regulatory Bateman domain of IMPDH can induce fluctuations between an expanded, active octamer and a collapsed, inactive octamer, both of which can be incorporated into filaments (26, 27). Previous studies demonstrated an association between deficiency in GMP synthesis and IMPDH filament formation. Early studies showed that IMPDH inhibitors, such as mycophenolic acid or ribavirin, cause rapid formation of IMPDH filaments in cultured cells (20, 22, 28). Depriving cells of essential purine precursors by limiting glutamine (29) or folate derivatives supplied by the thymidylate cycle (30) likewise cause IMPDH to polymerize. Glutamine deprivation and glutamine analogs have similar effects on the formation of CTPS filaments (31, 32). Remarkably, CTPS and IMPDH filaments can interact with each other in cells treated with 6-diazo-5-oxo-L-norleucine or 3′-deazauridine, suggesting the possibility of coordination between the two enzymes, but the implications of this observation remain unexplored (22, 33–35).

A few recent reports have provided new insights into how filament formation might regulate IMPDH activity. In the first study, 3′-deazauridine promoted IMPDH filament formation and led to an increased cellular GTP pool size, suggesting that IMPDH polymerization correlates with an increase in catalytic activity (34). Later, another study using novel IMPDH2 point mutants that block or promote polymerization concluded that polymerization itself does not affect enzyme activity, and that both active and inactive conformations of IMPDH2 can assemble into filaments (27). The most recent study demonstrated a correlation between IMPDH filament formation and rapid cell proliferation in mouse induced pluripotent stem cells (iPSCs) (36). Experiments using IMPDH2-mutant HeLa cell lines incapable of forming filaments also showed that when IMPDH levels are suppressed, formation of IMPDH filaments helps maintain normal cell proliferation, suggesting that polymerization acts to boost IMPDH activity (36). Despite these important studies, there remains a clear lack of consensus on the function of IMPDH filaments in cultured cells. Even more elusive is the role of IMPDH filaments *in vivo*. To date, observation of spontaneous IMPDH filament formation *in vivo* remains limited to mouse pancreatic β cells (34). Both ideas motivated us to search for a physiologic process in which IMPDH filaments might form.

Several years ago, our laboratory reported that IMPDH forms filaments in a high percentage of untreated, undifferentiated mouse embryonic stem cells (ESCs) cultured in rich medium (22). We hypothesized that, aside from ESCs, IMPDH filaments might assemble in other highly proliferative cell types. It is well-established that IMPDH is essential for proliferation (37) and that lymphocytes rely heavily upon *de novo* purine synthesis and IMPDH activity to undergo massive proliferation in response to antigenic challenge (38, 39). Recently, increasing interest has been placed on elucidating the metabolic reprogramming that occurs during T cell activation (40, 41). One essential aspect of proliferation is the upregulation of nucleotide synthesis, which is controlled by many transcription factors, namely Myc and Rb/E2F (42). Myc, which directly regulates the expression of IMPDH (43, 44), has also been identified as a master regulator of metabolic reprogramming during T cell activation (45), highlighting the importance of IMPDH in T cell metabolism. In this study, we demonstrate widespread *in vivo* IMPDH filament formation in splenic T and B cells, proliferating splenocytes, and thymocytes of normal, healthy mice. We then use both *ex vivo* primary human polyclonal T cell activation and *in vivo* mouse antigen-specific T cell activation experiments to show that IMPDH forms filaments during T cell activation. This is the first report of IMPDH polymerization as a novel aspect of T cell activation and the first to show extensive *in vivo* IMPDH filament formation in a variety of cell types. We propose that lymphocyte activation will be a useful model for further in-depth molecular studies of IMPDH filament function.

## Materials and Methods

### Mouse Tissue Preparation

Spleens and thymuses were harvested fresh from C57BL/6J (B6) mice of various ages from 10 days to 12 months old (indicated in each experiment) or B6.Ly5a mice (described below), then fixed immediately by immersion in 4% paraformaldehyde in PBS overnight at 4°C. Tissues were then washed in PBS 3 times for 10 min each, and submerged in 70% ethanol prior to standard processing and paraffin-embedding protocols. All animal experiments were performed using protocols reviewed and approved by the University of Florida Institutional Animal Care and Use Committee.

### Immunofluorescence on Mouse FFPE Tissue Sections

Formalin/paraformaldehyde-fixed, paraffin-embedded (FFPE) tissue sections of 4 μm thickness were incubated in a drying oven at 58°C for 2 h, and allowed to cool for 3 min at room temperature (RT). Sections were then deparaffinized and rehydrated using sequential incubations in the following solutions: xylenes for 20 min (2 × 10 min), 100% ethanol for 10 min (2 × 5 min), 95% ethanol for 10 min (2 × 5 min), 70% ethanol for 10 min, 50% ethanol for 5 min, H_2_O for 5 min. Heat-induced epitope retrieval was performed at 95°C for 20 min in IHC Select Citrate Buffer pH 6.0 (21545, Millipore Sigma, Burlington, MA). Slides were allowed to cool to RT in citrate buffer for 35 min, then washed once in H_2_O for 5 min and twice in PBS for 5 min each. Sections were blocked with Background Sniper blocking reagent (BS966, Biocare Medical, Pacheco, CA) for 15 min at RT. After washing in PBS once for 5 min, sections were incubated with primary antibody diluted in PBS with 0.05% Tween 20 (PBS-Tween) overnight at 4°C. Slides were then washed in PBS-Tween 3 times for 5 min each, and sections were incubated with secondary antibody in PBS-Tween for 1 h at RT. After washing again in PBS-Tween, slides were mounted and counterstained with 4′,6-diamidino-2-phenylindole (DAPI).

### *Ex vivo* Activation and Immunofluorescent Staining of Primary Human T Cells

PBMCs were isolated from fresh whole blood of healthy human donors (LifeSouth, Gainesville, FL) by density gradient centrifugation using Ficoll-Paque Plus (GE Healthcare, Marlborough, MA). Aliquots of cells were stored in liquid nitrogen until use. Cells were thawed and cultured at 37°C in a 5% CO_2_ incubator for 2 h in RPMI 1640 (Corning Inc., Corning, NY) supplemented with 5% heat-inactivated human AB serum (HP1022HI, Valley Biomedical, Winchester, VA), 2 mM L-glutamine (Corning), and 100 IU penicillin and 100 μg/ml streptomycin (Corning). After 2 h, cells in suspension were collected and passed through a 70 μm cell strainer (Corning). Cells were centrifuged, counted using trypan blue staining, and then seeded at a density of 1 × 10^6^ cells/ml in a 24-well plate in RPMI 1640 supplemented with 2 or 16 mM L-glutamine. PBMCs were left untreated or treated with 5 μg/ml PHA or ConA, or with 75 μl Human T-Activator CD3/CD28 Dynabeads per 1 ml medium. Cells were then incubated at 37°C in 5% CO_2_ for 72 h. After 72 h, cells were collected and centrifuged in a Shandon Cytospin 2 Centrifuge onto glass slides coated with poly-L-lysine (Sigma-Aldrich, St. Louis, MO). For cells treated with Dynabeads, beads were removed using a magnet prior to centrifugation. In certain experiments, fresh medium was also added 1 h prior to collecting cells for centrifugation. After centrifugation, cells were fixed using 4% paraformaldehyde in PBS for 15 min at RT and permeabilized with 0.1% Triton X-100 in PBS for 4 min. Cells were then incubated with primary antibody for 1 h at RT and secondary antibody for 45 min at RT, prior to mounting and counterstaining with DAPI. PHA (00-4977-03), ConA (00-4978-03), and Dynabeads (11131D) were purchased from Thermo Fisher (Waltham, MA).

### Ovalbumin-Specific CD4^+^ T Cell Adoptive Transfer and Flow Cytometry

CD4^+^ T cells specific to ovalbumin (OVA) were purified from the spleens of B6.Cg-Tg(TcraTcrb)425Cbn/J (B6.OT-II.CD90.1) mice (graciously provided by Dr. Stephen Schoenberger, La Jolla Institute for Allergy and Immunology, La Jolla, CA). 2 × 10^5^ CD4^+^ T cells were then adoptively transferred to B6.SJL-Ptprc^a^Pep3^b^/BoyJ (B6.Ly5a) recipients that were intraperitoneally immunized 24 h later with 50 ug of NP_16_-OVA in alum. Spleens were collected 6 days post-immunization. Half of each spleen was used for flow cytometry, while the other half was fixed, paraffin-embedded, and analyzed by immunofluorescence.

For flow cytometry, single cell suspensions from individual spleens were stained in 40 μl volume of PBS with 2% fetal bovine serum, using the following monoclonal antibodies purchased from BioLegend (San Diego, CA), BD Biosciences (San Jose, CA), or Thermo Fisher: anti-CD4 (1 μg/ml, BV711 conjugate, clone GK1.5), anti-CD44 (1.3 μg/ml, V500 conjugate, clone IM7), anti-CD90.1 (0.2 μg/ml, PE/Dazzle 594 conjugate, clone OX-7), anti-TCR Vβ 5.1, 5.2 (10 μg/ml, FITC conjugate, clone MR9-4), and anti-TCR Vα2 (1 μg/ml, PerCP/Cy5.5 conjugate, clone B20.1). Antigen-specific OT-II cells were gated as CD4^+^ TCR Vβ 5.1, 5.2^+^ TCR Vα2^+^ CD90.1^+^.

### Antibodies for Immunofluorescence Microscopy

Primary antibodies used for immunofluorescence in this study include: rabbit polyclonal anti-IMPDH2 (primary cells: 1:500 dilution, FFPE tissues: 1:200 dilution, 12948-1-AP, Proteintech, Chicago, IL), rat monoclonal anti-CD3 (primary cells and FFPE tissues: 1:100 dilution, clone CD3-12, Bio-Rad, Hercules, CA), rat monoclonal anti-CD19 (FFPE tissues: 1:50 dilution, clone 6OMP31, Thermo Fisher), mouse monoclonal anti-Ki-67 (primary cells: 1:400 dilution, clone B56, BD Biosciences), rat monoclonal anti-Ki-67 (FFPE tissues: 1:200 dilution, clone SolA15, Thermo Fisher), rat monoclonal anti-GL7 (FFPE tissues: 1:50 dilution, clone GL7, BioLegend), PE/Dazzle 594-conjugated mouse monoclonal anti-CD90.1 (1:20 dilution, clone OX-7, BioLegend), and PE/Dazzle 594-conjugated mouse IgG1 κ isotype control (1:20 dilution, clone MOPC-21, BioLegend). All secondary antibodies were purchased from Thermo Fisher and used at 1:400 dilution: Alexa Fluor 488-conjugated goat anti-rabbit IgG (A11034), Alexa Fluor 568-conjugated goat anti-rat IgG (A11077), and Alexa Fluor 568-conjugated goat anti-mouse IgG (A11004). All tissue staining was performed in parallel with staining of control sections with secondary antibody only (see examples in Supplementary Figure 1). All slides were mounted and counterstained with DAPI simultaneously using Vectashield HardSet Antifade Mounting Medium (H-1500, Vector Laboratories, Burlingame, CA). Images were captured with a Zeiss Axiovert 200M microscope equipped with a Zeiss AxioCam MRm camera using 10 × (0.50 NA), 20 × (0.75 NA), or 40 × (0.75 NA) objective**s** (Carl Zeiss Microscopy, Jena, Germany).

### Image Analysis and Statistics

Cell counting, quantification of cells with IMPDH filaments, and quantification of Ki-67-positive cells were performed manually (by S.J.C.) using the Cell Counter plugin included in the Fiji distribution of ImageJ (46). Data are presented as mean values plus or minus the standard error of the mean (S.E.M.). Results from independent groups were compared using two-way analysis of variance (ANOVA) or two-tailed Student's *t*-test where appropriate. Figures 4E,F show proportions of cells within a treated group and do not represent data from independent groups. Common statistical tests used to compare independent groups are not appropriate to analyze the outcome of these experiments. Further information on statistical tests used is included within figure legends where appropriate. *P-*values < 0.05 were considered significant. All statistical analyses were performed using GraphPad Prism version 7.03 for Windows (GraphPad Software, La Jolla, CA).

## Results

### IMPDH Filaments Are Abundant in Splenic T Cells and B Cells

**Figure 1:**
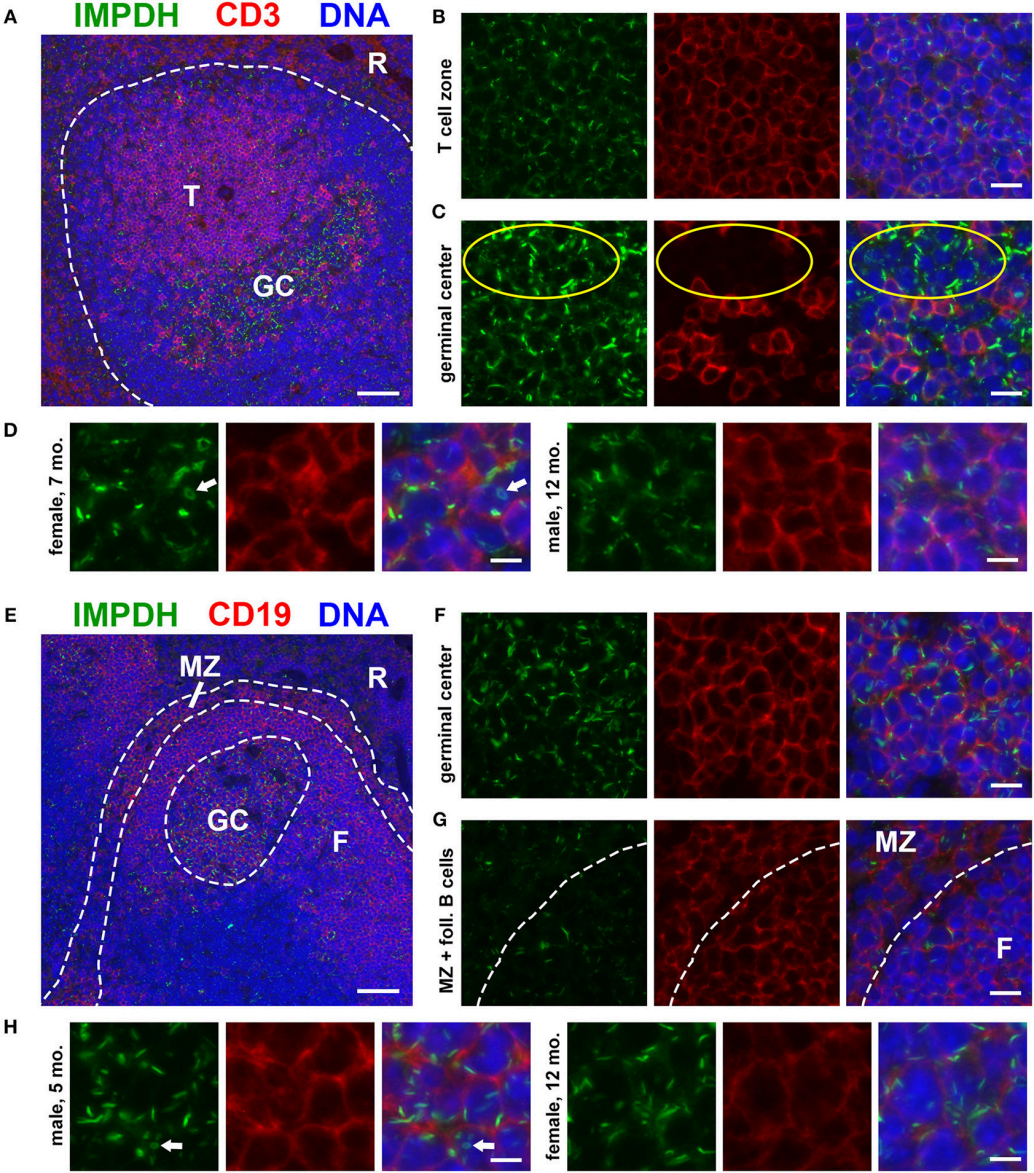
Splenic T and B cells form IMPDH filaments *in vivo*. **(A)** Representative image of a lymphoid follicle in an FFPE spleen section from an adult healthy B6 mouse stained for IMPDH (green) and T cell marker CD3 (red). Widespread IMPDH filament formation was detected throughout the spleen, including within the T cell zone (“T”), germinal centers (“GC”), other cells within the follicle, and even in the red pulp (“R”). **(B)** Representative image of the T cell zone showing many IMPDH filaments within T cells. **(C)** Representative image of a germinal center showing prominent IMPDH filaments in both CD3^+^ and CD3^−^ cells (highlighted by yellow circles). **(D)** Representative images of two different mice, a 7-months-old female and a 12-months-old male. Images were captured using the same objective as **(B,C)** but were enlarged 2× to more clearly show IMPDH filaments within T cells. White arrows: ring structure. **(E)** Lymphoid follicle from an adult healthy B6 mouse spleen stained for IMPDH (green) and B cell marker CD19 (red). IMPDH filaments were detected in GC B cells, follicular (“F”) B cells, and marginal zone (“MZ”) B cells. **(F)** Representative image of a germinal center depicting numerous IMPDH filaments within B cells. **(G)** Representative image illustrating IMPDH filament formation in follicular (“F”) and marginal zone (“MZ”) B cells. **(H)** Representative images of two different mice, a 5-months-old male and a 12-months-old female. Images were captured using the same objective as **(F,G)** but were enlarged 2× to more clearly show IMPDH filaments within B cells. White arrows: ring structure. Counterstain for all panels: DAPI (blue). Scale bars: 50 μm **(A,E)**, 10 μm **(B,C,F,G)**, 5 μm **(D,H)**.

To investigate the *in vivo* assembly of IMPDH filaments in highly proliferative cells, we first examined the spleens of normal, untreated mice for lymphocytes. We performed immunofluorescence on FFPE spleen sections from six adult B6 mice (three male, three female) of various ages, ranging from ~5 to 12 months old. Initially, we stained these spleens for IMPDH and T cell marker CD3. Remarkably, we consistently detected widespread IMPDH filament formation in many cells throughout the spleen, including CD3^+^ and CD3^−^ cells in both the white pulp and red pulp. However, due to high autofluorescence in the red pulp, we largely focused on lymphoid follicles (Figure 1A, representative image, red pulp marked “R”). We detected an abundance of IMPDH filaments throughout the T cell zone (“T” in Figure 1A) and germinal centers (GCs, determined by morphology and marked “GC” in Figure 1A). The T cell zone consists largely of T cells, a majority of which showed IMPDH filament formation (Figure 1B). GCs contain some follicular helper T cells (CD3^+^) but predominantly consist of activated and rapidly dividing B cells called centroblasts (CD3^−^). These cells give rise to centrocytes, which divide slowly and make up a small subset of germinal center cells. We were able to clearly detect IMPDH filaments in CD3^−^ cells (Figure 1C, yellow circles) next to CD3^+^ cells. As one may ascertain from images in Figures 1B,C, filaments within GCs consistently stained brighter and were more obvious, although not necessarily more numerous, than filaments in other areas of the tissue. However, the significance of this observation remains unclear and we were not able to quantify any difference in brightness or frequency of filaments between particular parts of the sections. Magnified images show that these filaments are relatively similar in size to many previous reports of IMPDH filaments (Figure 1D). We were also able to detect ring structures *in vivo*, which are a hallmark of IMPDH filaments (“rods and rings” structures) (Figure 1D, white arrow). Although it is unknown if there are any functional differences between rods and rings, the presence of rings indicated we were observing bona fide IMPDH filaments. Note that while we refer to these structures as IMPDH “filaments” in this study, we did not perform any high-resolution imaging capable of resolving individual filaments. Though they are likely the same, future experiments are required to confirm that IMPDH filaments reported in T cells are the same as filaments reported in other cell types (21, 23).

Having detected IMPDH filaments in CD3^−^ cells, we then stained these spleens for IMPDH and B cell marker CD19. Again, we identified abundant IMPDH filaments in B cells throughout the follicle (Figure 1E, representative image), including GC B cells (Figure 1F), follicular B cells (marked “F” in Figures 1E,G), and marginal zone B cells (“MZ” in Figures 1E,G). Magnified images clearly show rod- and ring-shaped structures in B cells (Figure 1H).

### IMPDH Filament Formation in Proliferating Cells of the Germinal Center

**Figure 2:**
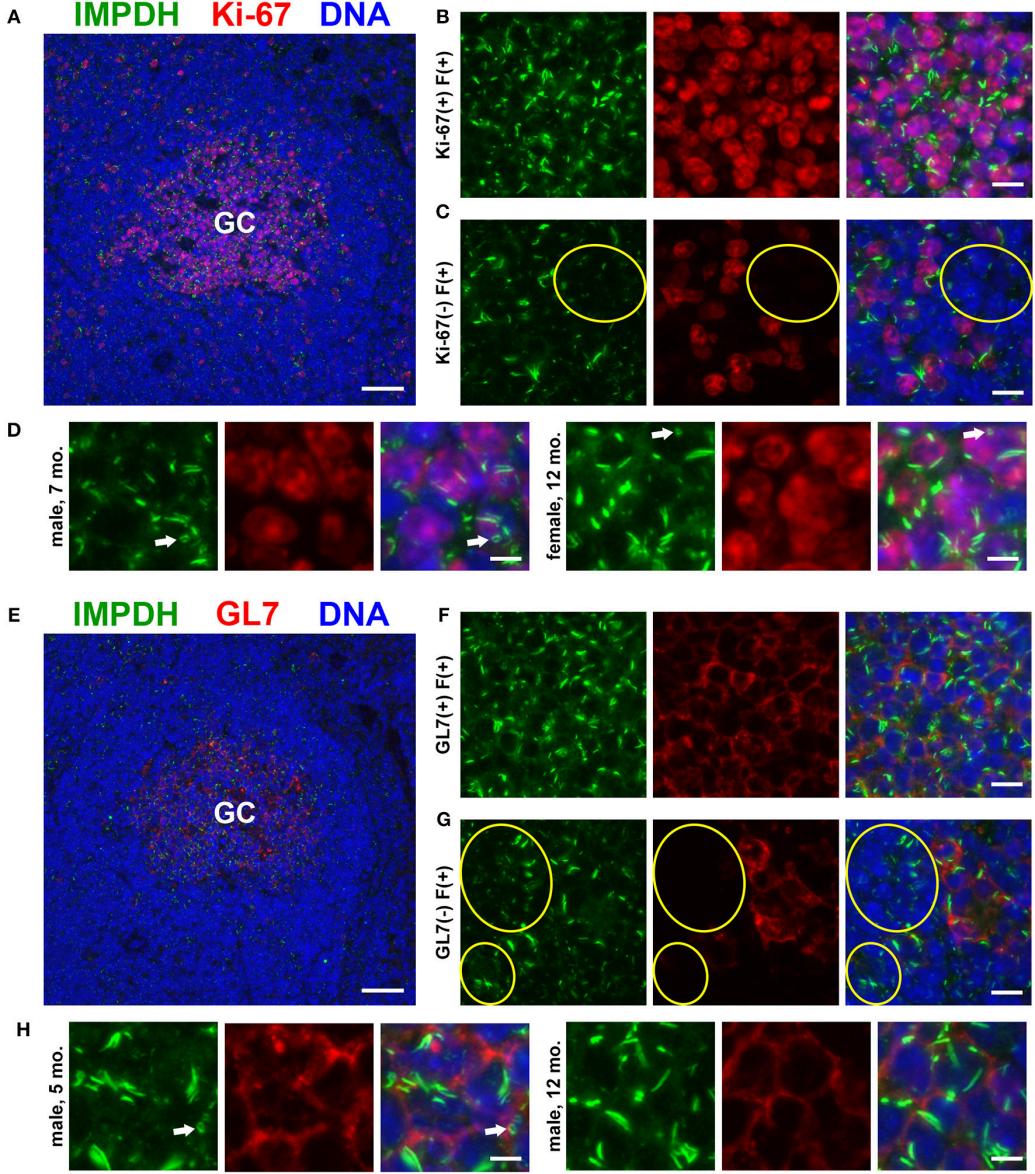
Proliferating cells in germinal centers form IMPDH filaments *in vivo*. **(A)** Representative image of a germinal center in an FFPE spleen section from an adult healthy B6 mouse stained for IMPDH (green) and proliferation marker Ki-67 (red). **(B)** Representative image of Ki-67-positive cells in GCs forming abundant IMPDH filaments. **(C)** Representative image showing that IMPDH filament formation was also commonly observed in Ki-67-negative cells (highlighted by yellow circles). **(D)** Representative images of two different mice, a 7-months-old male and a 12-months-old female. Images were captured using the same objective as **(B,C)** but were enlarged 2× to more clearly show IMPDH filaments within proliferating cells. White arrows: ring structures. **(E)** Serial section depicting the same GC as in **(A)**, but stained instead for IMPDH (green) and GC marker GL7 (red). **(F)** Representative image of extensive IMPDH filament formation by GL7^+^ cells. **(G)** Representative image showing that IMPDH filaments also form in GL7^−^ cells (marked by yellow circles). **(H)** Representative images of two different mice, a 5-months-old male and a 12-months-old male. Images were captured using the same objective as **(F,G)** but were enlarged2 × to more clearly show IMPDH filaments within GC cells. White arrows: ring structure. Counterstain for all panels: DAPI (blue). Scale bars: 50 μm **(A,E)**, 10 μm **(B,C,F,G)**, 5 μm **(D,H)**.

As our original hypothesis was that IMPDH filaments form in highly proliferative cells, we then stained the same spleen tissues for proliferation marker Ki-67, which is expressed in active stages of the cell cycle but not in resting cells (47). While sporadic Ki-67-positive cells with IMPDH filaments could be found throughout the spleen, the most obvious areas of Ki-67 expression were in GCs (Figure 2A). We detected IMPDH filaments in both Ki-67-positive (Figure 2B, magnified in Figure 2D) and Ki-67-negative cells (Figure 2C, yellow circles) within or near GCs. Finally, to confirm our morphological assessment of GCs, we stained more spleen sections for GL7, a commonly used marker for mouse GCs expressed on both B and T cells. Staining of serial sections demonstrated that Ki-67 staining matched up with GL7 staining (Figure 2E, same GC as shown in Figure 2A). Similar to previous experiments, we detected IMPDH filaments in both GL7^+^ cells (Figure 2F, magnified in Figure 2H) and GL7^−^ cells nearby (Figure 2G). Together, these data suggest that IMPDH filaments form in both T cells and B cells, especially proliferating GC cells likely to be centroblasts.

### Widespread IMPDH Filament Formation in Thymocytes of Young And Old Mice

**Figure 3:**
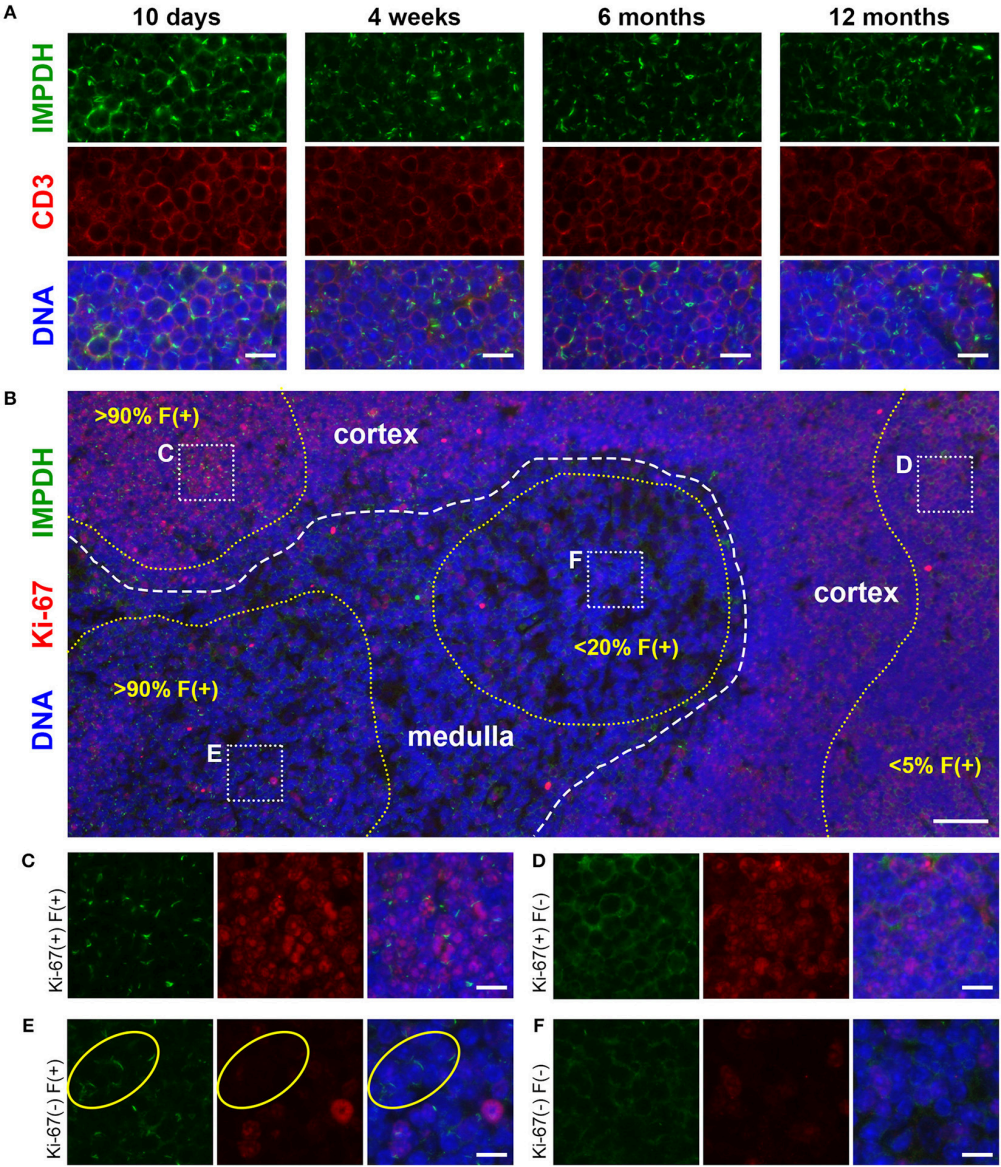
Cortical and medullary thymocytes of young and old mice form IMPDH filaments *in vivo*. **(A)** Representative images of FFPE thymuses from 10-days-old, 4-weeks-old, 6-months-old, and 12-months-old healthy B6 mice stained for IMPDH (green) and T cell marker CD3 (red). Widespread consistent IMPDH filament formation was observed in a majority of cells throughout the thymuses of mice at all ages. **(B)** Representative image demonstrating areas of the thymus with varying levels of IMPDH (green) filament formation and Ki-67 (red) expression. The dashed white line approximates the boundary between the cortex and medulla. As examples, percentage of cells with IMPDH filaments is written within areas outlined by yellow dotted lines. Ki-67 expression is clearly more prevalent in the cortex compared to medulla, as expected. Dotted white boxes labeled **(C–F)** correspond to **C-F** below. **(C–F)** Higher magnification images taken of the areas outlined in **(B)**. These representative images demonstrate four different cell populations in the thymus: **(C)** Ki-67-positive, filament-positive, **(D)** Ki-67-positive, filament-negative, **(E)** Ki-67-negative, filament-positive, and **(F)** Ki-67-negative, filament-negative. Yellow circles in **(E)** highlight the filament formation in Ki-67-negative cells. Counterstain for all panels: DAPI (blue). Scale bars: 10 μm **(A,C–F)**, 50 μm **(B)**.

After discovering the ability of mature T cells in the spleen to form IMPDH filaments, we turned to the thymus, the site of T cell maturation and large-scale proliferation of thymocytes (T cell precursors). Mechanisms controlling thymus development are generally conserved between humans and mice (48). Thymus volume increases relative to body size throughout the neonatal period, plateauing in post-natal life around 1 year in humans and within the first few weeks in mice (49). Thymus volume also directly correlates with the proliferation of thymocytes to produce a steady supply of naïve T cells (50). Accordingly, we started by examining 10-days-old (*n* = 2) and 4-weeks-old (*n* = 6) normal, untreated B6 mice in attempt to capture IMPDH filament formation during periods of the highest predicted thymocyte proliferation. We also examined the thymuses of the 6 mice in which we examined the spleen (Figures 1, 2), ranging from 5 to 12 months old. To our surprise, we consistently found an abundance of IMPDH filaments throughout the thymuses of mice from all age groups (Figure 3A, representative images). Although the bulk of thymocyte proliferation occurs in the thymic cortex, we were able to visualize large-scale filament formation in both the cortex and medulla. We turned again to Ki-67 staining to visualize proliferation and filament formation simultaneously. Surprisingly, a majority of cells within the thymus were positive for IMPDH filaments. However, there were clearly areas of tissue with differential filament formation. Figure 3B shows one such example, where neighboring areas of cortex and medulla both exhibited filament formation in >90% of cells. This dropped to < 20% in other areas of the medulla and even approached 0% in some areas of the cortex, despite widespread Ki-67 expression indicating that these cortical thymocytes were proliferating. Overall, we clearly detected 4 different cell populations in the thymus: (1) Ki-67-positive, filament-positive, (2) Ki-67-positive, filament-negative, (3) Ki-67-negative, filament-positive, and (4) Ki-67-negative, filament-negative (Figures 3C–F, representative images that correspond to dotted squares in panel B). As expected, the thymic cortex was overwhelmingly positive for Ki-67 expression and it was easy to find proliferating cortical thymocytes with IMPDH filaments (Figure 3C). Also as expected, far fewer proliferating cells were observed in the thymic medulla. However, across all age groups, we consistently detected areas of the medulla with an abundance of IMPDH filaments (Figures 3B,E). These data provide additional evidence that IMPDH filaments function in a variety of physiological processes involving rapidly proliferating cell types. That widespread IMPDH filament formation can be detected in non-proliferating cells also hints that the polymerization of IMPDH might be playing other important roles yet to be determined.

### IMPDH Forms Filaments During *ex vivo* Primary Human T Cell Activation

**Figure 4:**
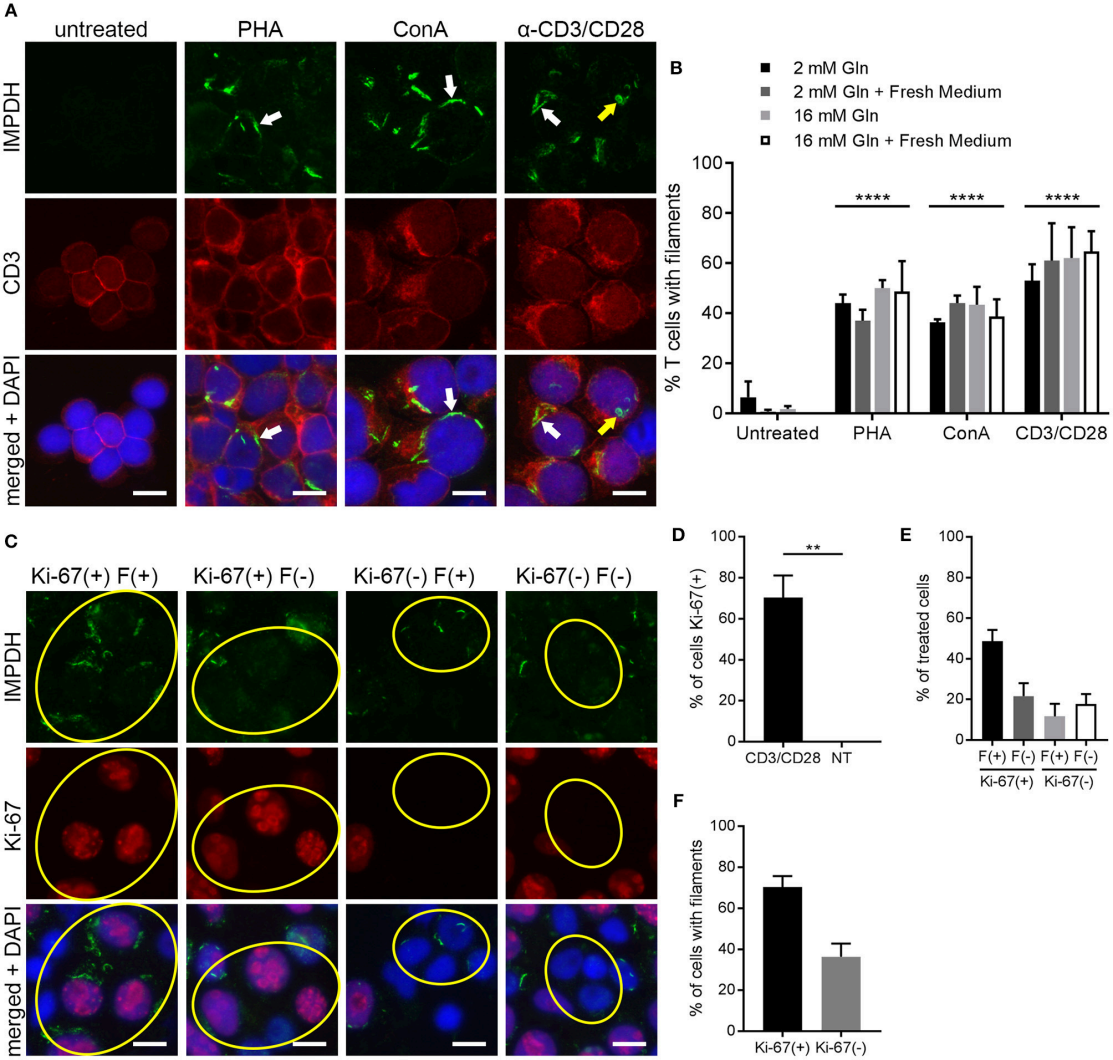
IMPDH forms filaments during *ex vivo* primary human T cell activation. **(A)** Representative images of T cells left untreated or stimulated by mitogens PHA, ConA, or anti-CD3/CD28 for 72 h, then fixed and stained for IMPDH (green) and T cell marker CD3 (red). White arrows: examples of IMPDH filaments. Yellow arrows: example of a ring-shaped filament. Images were captured using identical microscope settings for all treatment groups. **(B)** Quantification of the percentage of T cells that form filaments when untreated or treated with mitogens PHA, ConA, or anti-CD3/CD28. Cells were cultured in RPMI 1640 under four different conditions: 2 mM glutamine (Gln), 2 mM Gln + 1 h fresh medium, 16 mM Gln, or 16 mM Gln + 1 h fresh medium (represented by differently shaded bars). Different culture conditions were grouped together according to mitogenic treatment and compared to untreated cells (e.g., all PHA-treated conditions grouped vs. all untreated conditions grouped) and statistical significance displayed above each group. No significant differences were observed within these treatment groups due to culture conditions (e.g., PHA 2 mM Gln vs. PHA 16 mM Gln, no difference). Statistical test used: two-way ANOVA followed by Tukey's multiple comparisons test; ****p < 0.0001. Data are shown as mean ± S.E.M. and represent three independent experiments using 3 different human donors. **(C)** Representative images of PBMCs stimulated by anti-CD3/CD28 and stained for IMPDH (green) and proliferation marker Ki-67 (red). Yellow circles show examples of 4 different cell populations quantified in subsequent panels: Ki-67(+) filament(+), Ki-67(+) filament(–), Ki-67(–) filament(+), Ki-67(–) filament(–). **(D)** Quantification of the percentage of Ki-67-positive cells in anti-CD3/CD28-treated cells compared to untreated (NT) cells. Statistical test used: two-tailed Student's *t*-test; **p < 0.01. **(E)** Graph showing the proportion of anti-CD3/CD28-treated cells made up by the four different cell populations shown in **(C)** (all four groups add up to 100%). **(F)** Comparison of the percentage of treated cells with filaments between Ki-67-positive and Ki-67-negative cell populations from the experiment in **(C)**. **(E,F)** show proportions calculated from within the treated group only (anti-CD3/CD28-treated) and do not represent data from independent groups. Common statistical tests used to compare independent groups are not appropriate to analyze the outcome of these experiments, so no statistical significance is indicated. Data from **(D–F)** are shown as mean ± S.E.M. and represent 3 independent experiments. Counterstain for all panels: DAPI (blue). Scale bars: 10 μm.

To further probe and better quantify the IMPDH filament formation we observed in lymphocytes and thymocytes *in vivo*, we then examined *ex vivo* mitogenic stimulation of primary human T cells. We treated human PBMCs with the well-known mitogens PHA or ConA, or with monoclonal antibodies against CD3 and CD28 (anti-CD3/CD28) immobilized on the surface of microbeads. These microbeads mimic the interaction of an antigen-presenting cell with the T cell receptor-CD3 complex and co-stimulatory receptor CD28. In a preliminary experiment, PBMCs were cultured in RPMI 1640 medium with 2 mM glutamine and left untreated or incubated with PHA, ConA, or anti-CD3/CD28 for 72 h. Cells were then centrifuged onto glass slides, fixed, and stained with anti-IMPDH and anti-CD3 antibody to detect IMPDH filament formation in T cells. Our initial observation was that mitogen-stimulated T cells had a clear increase in IMPDH filament formation compared to untreated cells, which had almost no detectable filaments (Figure 4A). IMPDH signal was visibly lower in untreated compared to treated T cells, correlating with the significant increase in IMPDH expression post-stimulation reported in previous studies (39, 51, 52). However, we knew from previous studies that glutamine or serine deprivation can drive the formation of IMPDH filaments, which readily disassemble (in < 15 min) upon replenishment of glutamine or serine (29, 30). We also considered the substantial increase in glutamine uptake known to occur during T cell activation (45, 53, 54). Thus, our initial explanation was that these T cells might have formed IMPDH filaments because they depleted the culture medium of glutamine, serine, or another possibly unrelated metabolite over 3 days of stimulation. We then repeated our first experiment, except this time we also tested whether excess glutamine or adding fresh medium to the culture 1 h prior to harvesting the cells could disassemble the filaments. PBMCs were stimulated with PHA, ConA, or anti-CD3/CD28 and harvested after culture in RPMI 1640 for 72 h under 4 different conditions: (1) 2 mM glutamine, (2) 2 mM glutamine + 1 h fresh medium, (3) 16 mM glutamine, and (4) 16 mM glutamine + 1 h fresh medium. We observed a significant increase in the percentage of cells forming filaments in mitogen-stimulated compared to untreated T cells (Figure 4A, representative images; Figure 4B, quantification). Surprisingly, there were no significant differences among the four different culture conditions. Neither the addition of fresh medium nor the supply of excess glutamine had any impact on IMPDH filament formation. These data suggested to us that IMPDH filament formation is a genuine aspect of T cell activation that is not due to a nutrient deficiency in the culture medium.

### IMPDH Filament Formation Is Associated With Cell Proliferation

The data from the previous experiments indicated that after 72 h of stimulation, only roughly 40–60% of T cells were forming filaments. We also noted that IMPDH filaments formed in a very low percentage of untreated T cells. Clearly, there was not an absolute correlation between cell activation and filament formation. We then stimulated PBMCs again with anti-CD3/CD28 and stained them for the proliferation marker Ki-67. Within this population of stimulated cells, we observed 4 different subgroups: (1) Ki-67-positive cells with and (2) without filaments, and (3) Ki-67-negative cells with and (4) without filaments (Figure 4C, representative images, yellow circles highlight cells of each subgroup). Approximately 70% of anti-CD3/CD28-treated cells were Ki-67-positive compared to 0% of untreated cells (Figure 4D). We then quantitated what proportion of these treated cells belonged to each of the 4 different subgroups. On average, 49% were Ki-67-positive, filament-positive, 22% were Ki-67-positive, filament-negative, 12% were Ki-67-negative, filament-positive, and 18% were Ki-67-negative, filament-negative (Figure 4E). From these data, we also calculated that a higher percentage of proliferating cells formed filaments than non-proliferating cells. An average of 70% of Ki-67-positive cells contained filaments compared to just 36% of Ki-67-negative cells (Figure 4F). Overall, the data show a trend suggesting an association between IMPDH filament formation and cell proliferation.

### IMPDH Forms Filaments During *in vivo* Antigen-Specific T Cell Activation

After demonstrating IMPDH filament formation in *ex vivo* polyclonal T cell activation, we wondered if the same process also occurs during antigen-specific T cell activation *in vivo*. To do this, we employed a well-established experimental model of antigen-specific T cell activation using B6.OT-II.CD90.1 mice, which carry CD4^+^ T cells with an MHC class II-restricted T cell receptor specific for ovalbumin (OVA) (55, 56). OVA-specific CD4^+^ T cells that express the CD90.1 allele were purified from the spleens of B6.OT-II.CD90.1 mice and adoptively transferred into B6.Ly5a recipient mice (expressing the CD90.2 allele), which were then immunized 24 h later with NP_16_-OVA in alum (*n* = 3). These adoptively transferred T cells can be identified by their cell surface expression of CD90.1, which is not expressed in the recipient mice. Spleens were collected 6 days post-immunization, then cut in half. One half was fixed in 4% paraformaldehyde prior to processing and embedding, while the other half was used for flow cytometry.

**Figure 5:**
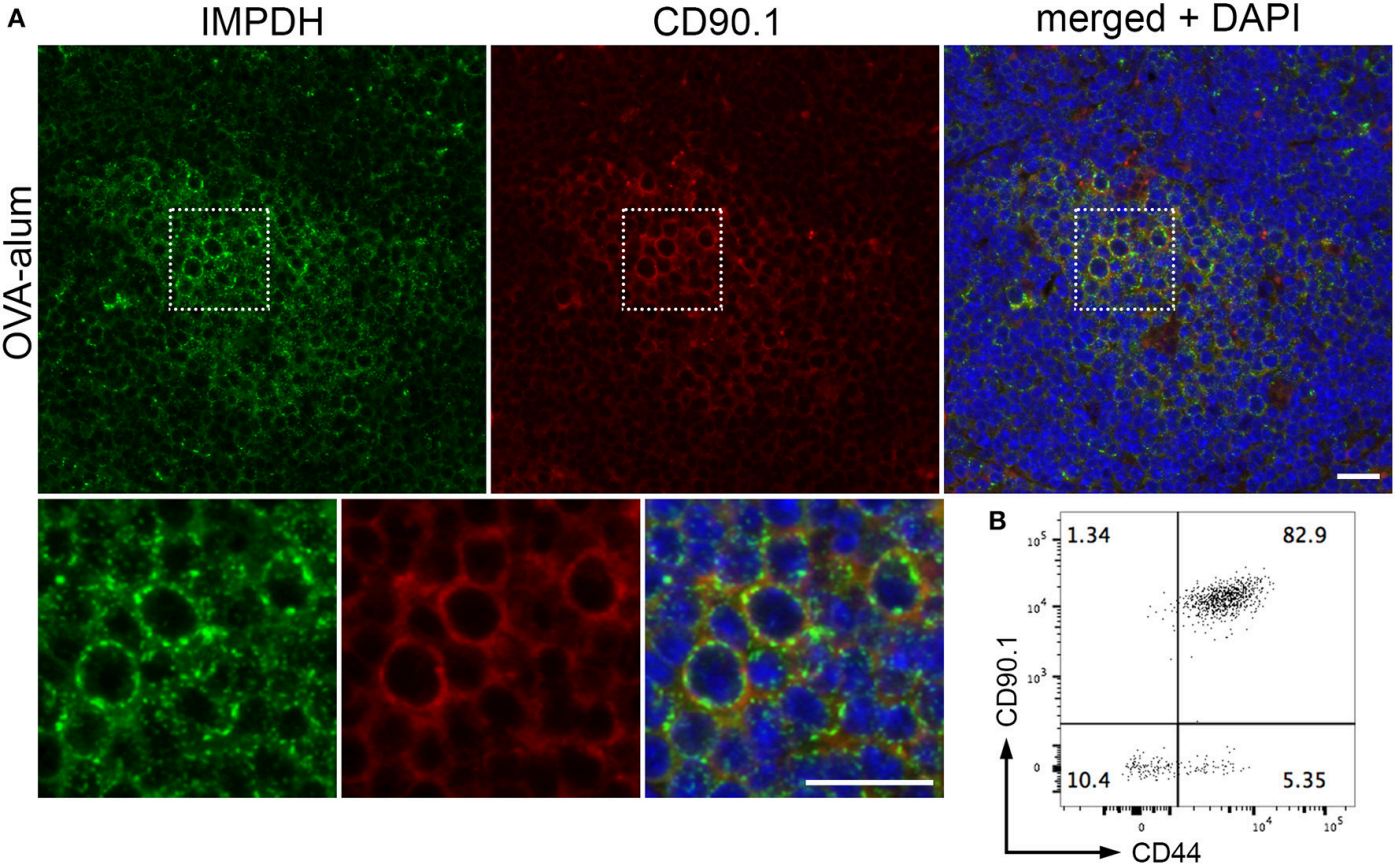
IMPDH forms filaments during *in vivo* antigen-specific T cell activation. **(A)** Representative images of OVA-specific adoptively transferred CD90.1^+^ (red) T cells with IMPDH (green) filaments in the spleen from a mouse immunized with OVA-alum. Areas marked by dotted squares are enlarged 3× and shown below to more clearly demonstrate IMPDH filaments in antigen-specific T cells. Counterstain: DAPI (blue). Scale bars: 20 μm. **(B)** Representative flow cytometry plot showing that a majority of adoptively transferred OVA-specific T cells (CD90.1^+^) are activated (CD44^+^) after immunization with OVA. Numbers indicate percentage of cells belonging to each quadrant.

By immunofluorescence, we were able to detect IMPDH filament formation in the transferred OVA-specific (CD90.1^+^) cells in spleens from all 3 mice (Figure 5A, representative images). In parallel, we used flow cytometry to confirm that these transferred T cells were properly activated upon OVA challenge. In all cases, a clear majority of OVA-specific T cells were positive for CD44, a marker for T cell activation (Figure 5B, representative plot). In addition, the weak staining of the CD90.1 antibody in immunofluorescence prompted us to use serial sections to compare CD90.1 staining to that of no antibody (Supplementary Figure 2A). We also compared CD90.1 staining to that of an isotype control antibody (Supplementary Figure 2B), concluding that we were observing a true positive signal for CD90.1. Importantly, this experiment demonstrates that, in addition to *ex vivo* human T cell activation, IMPDH filaments form during *in vivo* antigen-specific T cell activation.

## Discussion

Since the discovery that IMPDH is amplified in tumors and rapidly proliferating tissues (37), IMPDH has been an important target for the development of anticancer, antiviral, antibacterial, and immunosuppressive therapies (17, 57). IMPDH inhibitors have been used in clinics around the world for many years now, including azathioprine, mycophenolic acid, and mizoribine for immunosuppression and autoimmune diseases, and ribavirin for viral infections. Despite the long-term and widespread use of these drugs, it was not reported until around 10 years ago that treatment with IMPDH inhibitors causes a striking rearrangement of IMPDH protein into micron-scale filaments within cultured cells (20, 28). A few years later, our laboratory and others determined that a subset of hepatitis C patients, once exposed to ribavirin and interferon-α therapy, produce autoantibodies recognizing these IMPDH filaments, also referred to as “rods and rings” structures (22, 58, 59). These anti-rods/rings autoantibodies were not detected in the same patients prior to therapy, suggesting the possibility that ribavirin exposure alters the immunogenicity of IMPDH and leads to drug-induced autoantibody generation (60, 61). Pharmacological doses of several other widely-used drugs that do not directly target IMPDH have been shown to cause IMPDH filament formation in cultured cells, including pemetrexed (non-small cell lung cancer, mesothelioma), methotrexate (various cancers, autoimmune diseases), and acyclovir (herpes, shingles) (30, 62, 63). These data suggest the likelihood of widespread IMPDH filament assembly in the tissues of patients taking these drugs. Indeed, IMPDH filaments have been observed in the PBMCs of patients treated with mycophenolic acid, ribavirin, azathioprine, and methotrexate (62). The clinical implications of both IMPDH filament formation and the autoantibody response to these structures remain largely unexplained, highlighting the need to better understand all aspects of IMPDH regulation.

Until recently, much of the work on IMPDH filaments focused on elucidating the subcellular conditions that drive polymerization of IMPDH. Aside from IMPDH inhibitors, it was shown early on that the GMP synthase inhibitor decoyinine (20) and glutamine analogs 6-diazo-5-oxo-L-norleucine and acivicin also lead to IMPDH filament formation (22). We then showed that cells cultured in glutamine-deficient medium gradually form IMPDH filaments over a few days, an effect that is exacerbated by addition of methionine sulfoximine, a glutamine synthetase inhibitor (29). Later, we also found that serine deprivation leads to IMPDH filament assembly with similar kinetics (30). In that same study, we demonstrated that inhibition or transient knockdown of serine hydroxymethyltransferase or dihydrofolate reductase, key enzymes in the thymidylate cycle of one-carbon metabolism, also promote IMPDH polymerization. In most of these subcellular conditions, IMPDH filaments readily disassemble upon addition of guanosine, implying a correlation between filament formation and deficiency in GMP synthesis. However, IMPDH filaments are not always clearly associated with inhibition of GMP synthesis. Our laboratory also discovered IMPDH filament formation in a high percentage of untreated, undifferentiated mouse ESCs cultured in rich medium (22). We later discovered IMPDH filaments also form in a low percentage of Ptk2, NIH/3T3, NRK, and RAW264.7 cells in rich media with no obvious manipulation (63). Similar observations have been made in CHO cells (64). Overall, these data comprise much of what was known about IMPDH filaments until just a couple years ago.

A few more recent studies from other laboratories have explored the effects of polymerization on the activity of IMPDH, using experimental models where IMPDH filaments form in the absence of any inhibitors that might alter IMPDH activity. In 2015, the Liu and Sung laboratories showed that the CTPS inhibitor 3′-deazauridine promotes IMPDH filament formation and leads to an increased cellular GTP pool size, suggesting that IMPDH polymerization correlates with an increase in catalytic activity (34). In the same study, IMPDH filaments formed *in vivo* in untreated mouse pancreatic β cells and polymerization decreased in mice fasted overnight, possibly correlating with nutrient uptake. In 2017, the Peterson and Kollman laboratories generated IMPDH2 point mutants that block or promote polymerization without affecting enzyme activity (27). They also demonstrated that both active and inactive conformations of IMPDH2 can be incorporated into filaments, concluding that polymerization itself does not affect IMPDH activity. Most recently, the Liu and Sung laboratories explored the function of IMPDH filaments in mouse iPSCs and CRISPR/Cas9-edited IMPDH2-mutant HeLa cell lines with no filament-forming capability (36). Their data suggest that the polymerization of IMPDH is modulated by the ratio of its substrate IMP to guanine nucleotides, and that filament formation acts to increase IMPDH activity during periods of high demand for guanine nucleotides. These studies demonstrate that although significant progress has been made, there is still no consensus on the function of IMPDH filaments. These recent data also call to mind other reports that enzymes in *de novo* purine synthesis can assemble into a dynamic metabolic complex termed the purinosome (65). Purine depletion drives purinosome assembly, which acts to increase *de novo* synthesis rates of IMP, GMP, and AMP (66). Remarkably, GFP-tagged adenylosuccinate synthase and IMPDH co-localized with OFP-tagged formylglycinamidine ribonucleotide synthase (purinosome marker) in purine-depleted media, suggesting that IMPDH is also incorporated into purinosomes (66). Although they appear to be distinct structures, the relationship between purinosomes and IMPDH filaments and their potentially synergistic functions in proliferation warrant further investigation.

In the present study, we were motivated by the different (yet not necessarily conflicting) data regarding the function of IMPDH filaments, as well as the lack of *in vivo* data on IMPDH filament formation. We thought it prudent to seek a physiological process in which IMPDH filaments might play a role. Considering our previous data showing extensive IMPDH polymerization in stem cells (22), we wondered if other highly proliferative cell types might form filaments “naturally” (i.e., without inhibitors or nutrient deprivation). Since *de novo* purine synthesis and IMPDH activity are critical for the rapid proliferation of T cells in the immune response (18, 38, 39), we thought this cell type an obvious choice for further investigation. As the data show, IMPDH filament formation correlates with the proliferation of T cells stimulated by classic mitogens, PHA and ConA, as well as anti-CD3 and anti-CD28 antibodies that mimic physiological antigen presentation by antigen-presenting cells. These observations also extend beyond *ex vivo* mitogenic stimulation to *in vivo* antigen-specific T cell activation. These data agree with previous data from our laboratory that show undifferentiated mouse ESCs form abundant IMPDH filaments, which disassemble once cells are differentiated and stop proliferating (22). Our present data also agree with recently published data showing that spontaneous (natural) formation of IMPDH filaments in iPSCs correlates with rapid cell proliferation (36). In recent years, there has been increasing interest in understanding the dramatic shifts in cellular metabolism between resting cells and proliferating cells, referred to as metabolic reprogramming. It has been known for some time that tumor cells carry out glycolysis despite the presence of oxygen, also known as the Warburg effect (67). The same metabolic phenotype has been observed in lymphocytes (68–71) and stem cells (72, 73), suggesting that these changes might represent a general metabolic program of proliferating cells. A very recent study also demonstrated that distinct metabolic signatures can be detected in different subsets of developing thymocytes (74). Although our data are limited in that we do not know if IMPDH filament formation differs between thymocyte subsets, it may explain why we observed varying levels of IMPDH filaments throughout the thymus. The significant increase in biomass accumulation and cell division rate during lymphocyte proliferation relies heavily on *de novo* synthesis to generate sufficient nucleotide levels (45, 75). IMPDH filament formation might represent a novel aspect of lymphocyte activation that helps meet the heightened demand for guanine nucleotides. Further experiments will elucidate where IMPDH filament formation fits into the chronology of metabolic reprogramming that occurs during T cell activation and stem cell proliferation.

What was perhaps even more striking to us than discovering IMPDH filaments in T cell activation was the widespread IMPDH filament formation in different cell types of healthy mouse spleen and thymus. Because a previous study demonstrated IMPDH filament formation specifically in mouse pancreatic β cells, and no other cell types (34), we speculated that spontaneous *in vivo* IMPDH filament formation might be restricted to specific cell types or tissues and could be hard to detect. While our data generally support the notion of a correlation between IMPDH polymerization and cell proliferation, this might not tell the whole story. Both *ex vivo* human and *in vivo* mouse data indicate that, in general, expression of the proliferation marker Ki-67 correlates with IMPDH filament formation. The expression of Ki-67 protein is strictly associated with active phases of the cell cycle (G1, S, G2, mitosis) and is absent in resting cells (G0) (47). However, in both human primary cells and mouse tissues, we clearly observed Ki-67-positive cells both with or without IMPDH filaments, as well as Ki-67-negative cells both with or without IMPDH filaments. One possible explanation for Ki-67-negative, filament-positive cells could be the stability of IMPDH filaments. Perhaps IMPDH that polymerizes during an active proliferation cycle might remain polymerized when a cell exits the cell cycle into a resting state. For Ki-67-positive, filament-negative cells, it is possible that IMPDH filaments are not formed as early as Ki-67 expression begins. For example, if a cell exits G0 and enters G1, Ki-67 expression might begin earlier in G1 prior to a high demand for guanine nucleotides (when IMPDH filaments would form) that occurs in late G1 (42). Both explanations are supported by recently published data from a thymidine block experiment where mouse iPSCs were analyzed for EdU incorporation and IMPDH filament formation at different time-points post-block (36). At 4 h post-thymidine, 0% of cells were EdU-positive, yet IMPDH filaments were still present in ~35% of cells. At 12 h, once 0% of cells were EdU-positive or filament-positive, thymidine was removed and dCTP added to restore proliferation. After 3 h, ~64% of cells were EdU-positive, yet only ~21% of cells contained filaments. IMPDH filament formation did not recover to normal levels (~82% of cells) until 15 h after dCTP addition. These data support the hypothesis that IMPDH filaments might remain stable in cells no longer proliferating, and that their formation might lag behind other markers once proliferation is restored.

It is counterintuitive that IMPDH filaments naturally form in highly proliferative cells, yet IMPDH inhibitors that arrest proliferation cause similar filament formation. Many IMPDH inhibitors, like azathioprine, mycophenolic acid, and mizoribine, are used clinically as immunosuppressants, limiting lymphocyte proliferation. This complicates the observation that IMPDH filaments are found in rapidly proliferating cells, like lymphocytes. An alternative hypothesis is that IMPDH filaments are not exclusively linked to proliferation and that other factors contribute to their assembly and disassembly. For years, we considered that since IMPDH filament formation is associated with deficiency in GMP synthesis, perhaps the filaments are composed of largely inactive enzyme. Several studies have revealed that inhibiting essentially any enzyme upstream or downstream of IMPDH in *de novo* purine/GMP synthesis or the thymidylate cycle of one-carbon metabolism leads to polymerization of IMPDH (20, 22, 28–30, 62, 63). When treating cells with IMPDH inhibitors, which cause G1 arrest as well as very rapid (5–10 min) and extensive IMPDH polymerization, the majority of cellular IMPDH is expected to be inactive. When inhibiting upstream of IMPDH, as with methotrexate treatment, the situation is not as clear. While substrate depletion caused by inhibition of *de novo* IMP synthesis would render IMPDH mostly inactive, salvage of hypoxanthine by hypoxanthine-guanine phosphoribosyltransferase can replenish IMP to some degree, depending on the cell type. However, it is known that methotrexate treatment causes an irreversible G1/S arrest that is mediated, at least partially, through depletion of purines that is not overcome by salvage (76–80). IMPDH filaments also persist in >90% of HeLa cells treated with pharmacological doses of methotrexate for at least 24 h (30), which should be well after cell cycle arrest occurs. Thus, IMPDH filaments formed after treatment with IMPDH inhibitors or methotrexate should not be considered the same as filaments in proliferating cells. It might be that, once these inhibitors are added to cells, an immediate need for more GTP stimulates the rapid formation of filaments. However, the continued presence of inhibitor will likely maintain a predominantly inactive pool of IMPDH within the filaments. As mentioned, it was shown recently that both active and inactive IMPDH octamer conformations can form filaments *in vitro* (27). In living cells, it remains unclear what molecular mechanism drives the polymerization of IMPDH. This mechanism might be common to both active and inactive octamers.

We are encouraged that future experiments using T cell activation as a model for IMPDH filament formation will help answer some of the many questions still surrounding the polymerization of IMPDH. Despite the progress in developing IMPDH inhibitors for immunosuppressive and antiviral therapies, less success has been found in specific targeting of IMPDH in cancers. Several recent studies have demonstrated increased expression of IMPDH in bladder, kidney, nasopharyngeal, prostate, and small cell lung cancers (81–84), highlighting the necessity to better understand all aspects of IMPDH regulation, including polymerization. Based on the data presented here and other recently published work, it is conceivable that manipulating the ability of IMPDH to polymerize could be exploited in the design of novel therapies. We have already recognized that widely-used drugs, like methotrexate, mycophenolic acid, and ribavirin, profoundly alter the subcellular distribution of IMPDH in the cells of patients, with unknown consequences. From the present study, we now also know that extensive IMPDH filament formation occurs in the spleen and thymus (and perhaps other proliferative tissues) of healthy mice. Importantly, our data suggest that IMPDH filament formation is a novel and largely uncharacterized aspect of T cell activation. Better understanding of IMPDH filament function in this process could have widespread impacts on a variety of treatment regimens, especially the many emerging T cell-based immunotherapies. These ideas should inspire intense interest in this enzyme.

## Ethics Statement

This study was carried out in strict accordance with the recommendations in the Guide for the Care and Use of Laboratory Animals of the Animal Welfare Act and the National Institutes of Health guidelines for the care and use of animals in biomedical research. All animal protocols were approved by the Institutional Animal Care and Use Committee (IACUC) of the University of Florida, Gainesville (OLAW Assurance # A3377-01).

## Authors' Note

While this manuscript was under review, Duong-Ly et al. published an elegant study demonstrating IMPDH filament formation in *ex vivo* activation of healthy murine splenic T cells, as well as in T cells isolated from mice infected with lymphocytic choriomeningitis virus. They showed that filament assembly was diminished by mTOR inhibitors or knockout of the Ca^2+^ influx regulator STIM1 (85).

## Author Contributions

SC conceived the study, designed and performed experiments, analyzed the data, and wrote the original draft of the manuscript. GA designed and performed experiments. HK performed experiments. LM designed experiments. EC conceived the study, analyzed the data, and critically reviewed and edited the manuscript. All authors read and approved the final manuscript.

### Conflict of Interest Statement

The authors declare that the research was conducted in the absence of any commercial or financial relationships that could be construed as a potential conflict of interest.

## Abbreviations

AMP: adenosine monophosphate
ConA: concanavalin A
CTPS: cytidine triphosphate synthase
dCTP: deoxycytidine triphosphate
EdU: 5-ethynyl-2′-deoxyuridine
ESCs: embryonic stem cells
FFPE, formalin/paraformaldehyde-fixed: paraffin-embedded
GC: germinal center
GMP: guanosine monophosphate
GTP: guanosine triphosphate
IMP: inosine monophosphate
IMPDH: inosine monophosphate dehydrogenase
iPSCs: induced pluripotent stem cells
OVA: ovalbumin
PBMCs: peripheral blood mononuclear cells
PE: phycoerythrin
PHA: phytohemagglutinin
