# Supplementary material for: Immune Response-Dependent Assembly of IMP Dehydrogenase Filaments
Source: Front Immunol. 2018 Nov 29;9:2789. doi: 10.3389/fimmu.2018.02789 (PMC6283036; doi:10.3389/fimmu.2018.02789)
Supplement: Supplementary file 1 [file Data_Sheet_1.PDF]

Supplementary Material

Immune response-dependent assembly of IMP dehydrogenase filaments

S. John Calise, Georges Abboud, Hideko Kasahara, Laurence Morel, and Edward K.L. Chan*

*** Correspondence:** Corresponding Author: echan@ufl.edu

1.1 Supplementary Figures

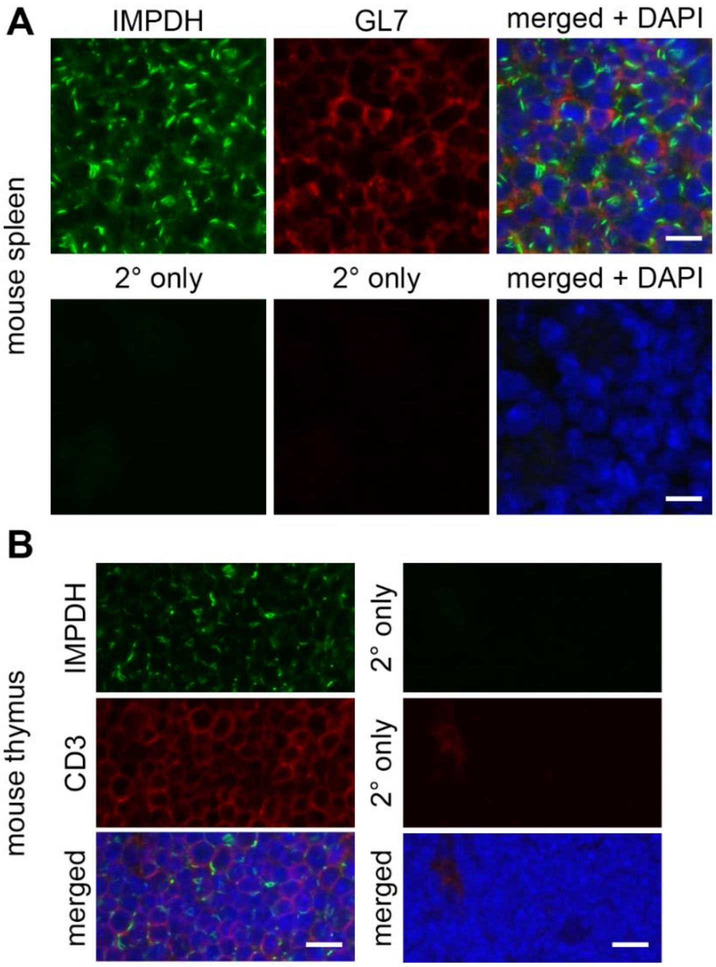

Supplementary Figure 1. Example images of secondary-only controls used for tissue staining. (A) Fig. 2F is reproduced here and compared to a representative image of a mouse spleen stained with secondary antibodies only (primary antibodies omitted from staining procedure). **(B)** Images labeled "6 months" from Fig. 3A are reproduced here and compared to a representative image of a mouse thymus stained with secondary antibodies only. Images for controls were taken using comparable exposure times. Counterstain for all panels: DAPI (blue). Scale bars: 10 µm.

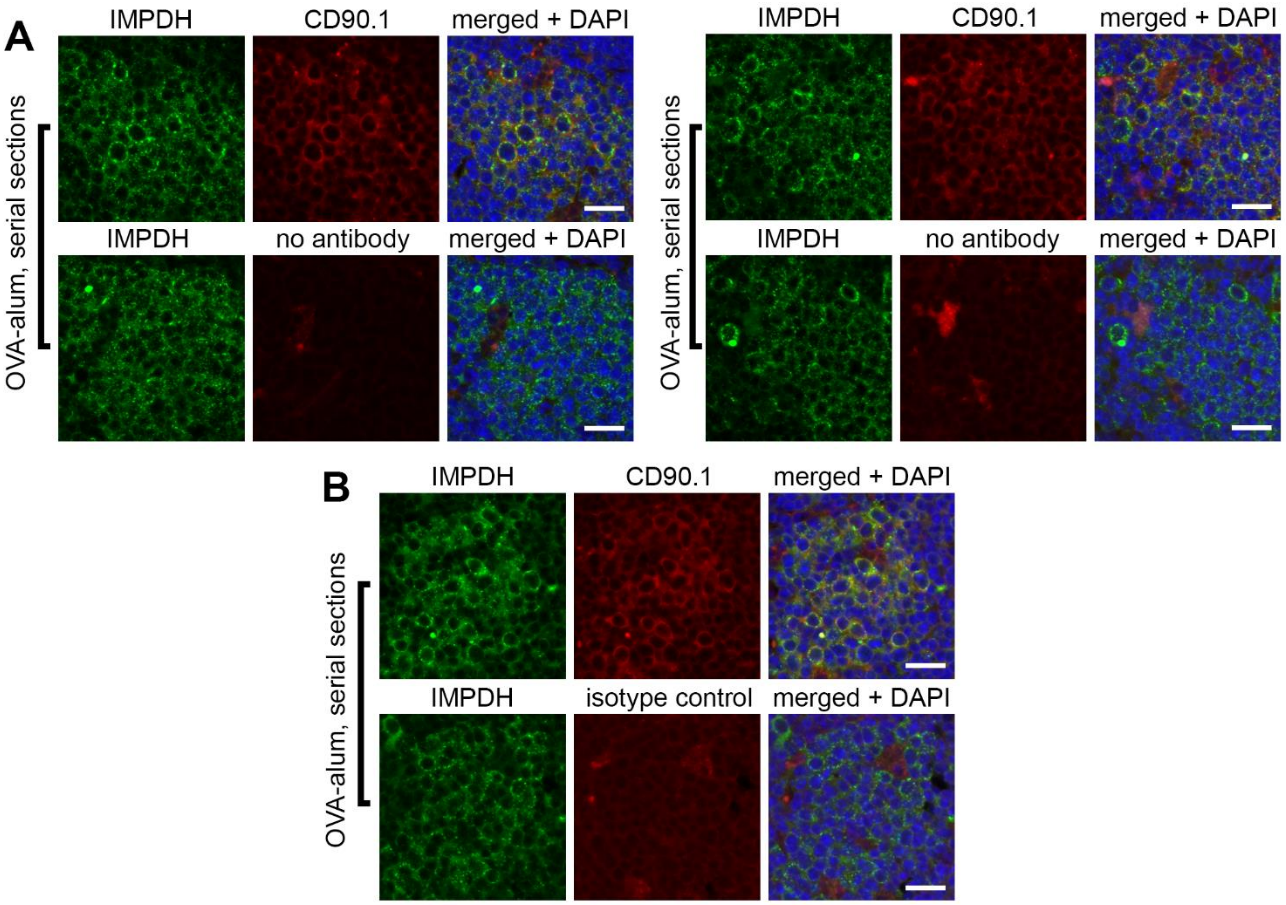

Supplementary Figure 2. Confirmation of true positive CD90.1 signal in mice with adoptively transferred CD4$^+$ CD90.1$^+$ T cells. (A) Top rows: representative images of adoptively transferred OVA-specific CD90.1$^+$ (red) cells with IMPDH (green) filaments in spleens from mice immunized with OVA-alum. Bottom rows: serial sections of the same areas of tissue, except CD90.1 antibody was omitted from the staining procedure. **(B)** Similar experiment to panel A, except CD90.1 staining was compared to staining of an isotype control antibody that does not recognize any antigen. Counterstain for all panels: DAPI (blue). Scale bars: 20 µm.
